# Transdiagnostic phenomena of psychopathology in the context of the RDoC: protocol of a multimodal cross-sectional study

**DOI:** 10.1186/s40359-023-01335-8

**Published:** 2023-09-28

**Authors:** Marvin S. Meiering, David Weigner, Sören Enge, Simone Grimm

**Affiliations:** 1 Department of Natural Sciences, MSB Medical School Berlin, Rüdesheimer Straße 50, 14197 Berlin, Germany; 2 Department of Education and Psychology, Freie Universität Berlin, Habelschwerdter Allee 45, 14195 Berlin, Germany; 3 Department of Psychiatry and Psychotherapy, Charité- Universitätsmedizin Berlin, corporate member of Freie Universität Berlin and Humboldt-Universität Zu Berlin, Hindenburgdamm 30, 12203 Berlin, Germany; 4 Department of Psychiatry, Psychotherapy and Psychosomatics, Psychiatric University Hospital Zurich, University of Zurich, Lenggstrasse 31, CH-8032 Zurich, Switzerland

**Keywords:** Neuroticism, Hedonic capacity, Rumination, Negativity bias, Emotion regulation, Personality, Psychopathology, Healthy volunteers, Functional magnetic resonance imaging, Research Domain Criteria (RDoC)

## Abstract

In the past, affective and cognitive processes related to psychopathology have been examined within the boundaries of phenotype-based diagnostic labels, which has led to inconsistent findings regarding their underlying operating principles. Investigating these processes dimensionally in healthy individuals and by means of multiple modalities may provide additional insights into the psychological and neuronal mechanisms at their core. The transdiagnostic phenomena Neuroticism and Rumination are known to be closely linked. However, the exact nature of their relationship remains to be elucidated. The same applies to the associations between Hedonic Capacity, Negativity Bias and different Emotion Regulation strategies.

This multimodal cross-sectional study examines the relationship of the transdiagnostic phenomena Neuroticism and Rumination as well as Hedonic Capacity, the Negativity Bias and Emotion Regulation from a RDoC (Research Domain Criteria) perspective. A total of 120 currently healthy subjects (past 12 months) will complete several questionnaires regarding personality, emotion regulation, hedonic capacity, and psychopathologies as well as functional magnetic resonance imaging (fMRI) during cognitive and emotional processing, to obtain data on the circuit, behavioral and self-report level.

This study aims to contribute to the understanding of the relationship between cognitive and affective processes associated with psychopathologies as well as their neuronal correlates. Ultimately, a grounded understanding of these processes could guide improvement of diagnostic labels and treatments. Limitations include the cross-sectional design and the limited variability in psychopathology scores due to the restriction of the sample to currently healthy subjects.

## Introduction

Mental disorders, especially internalizing disorders, are one of the leading causes of disability, affecting one in eight individuals worldwide, with numbers still rising [1–5] and therefore posing a significant public health issue. Currently, mental disorders are classified using phenomenologically based taxonomies such as the ICD or DSM [6, 7]. Although this approach has been useful in the past, patients falling under the same diagnostic label can show highly heterogenous symptoms and responses to the same treatment. Accordingly, new approaches attempting to tap into the neurobehavioral entities and mechanisms underlying mental disorders have been developed to provide more informative diagnostic labels and improve treatments.

The Research Domain Criteria initiative (RDoC [8, 9];), founded by the National Institute of Mental Health, provides a framework to investigate psychopathology as varying degrees of dysfunction in distinct psychological and biological systems. Namely the Positive Valence Systems, Negative Valence Systems, Cognitive Systems, Arousal and Regulatory Systems, Sensorimotor Systems and Social Processes constitute the general neurobehavioral domains of the RDoC. Each of the systems is in turn distinguished by so called (sub)constructs. The heterogenous and complex phenomenology of mental disorders is assumed to be driven by different degrees of aberrancy in these systems and their respective (sub)constructs. Investigating the operating principles of these systems dimensionally and across multiple modalities is at the core of RDoC, and accounts for the various sources of variability in findings regarding mental disorders and neurobehavioral functioning in general.

As the RDoC delineates a thorough framework to investigate neurobehavioral function in distinct systems through multiple units of analysis ranging from the level of genetics to self-reports, RDoC inspired research promises comprehensive insights into the underlying mechanisms associated with psychopathology. Applying the RDoC approach to the study of not only patients but also healthy volunteers (HV), may provide additional insight into these mechanisms. This RDoC inspired multimodal cross-sectional study of Positive Valence Systems, Negative Valence Systems and Cognitive Systems aims to elucidate the underlying mechanisms of transdiagnostic phenomena on the circuit, behavioral and self-report level in healthy subjects.

### Negative valence systems: interaction of neuroticism and rumination

#### Neuroticism

The Negative Valence Systems (NVS; [8, 10]) domain of the RDoC matrix encompasses the neurobehavioral systems centrally involved in the response to (potential) punishment and aversive situations or contexts in general. Several heritable and transtemporally stable dispositional traits are known to correspond to that definition of the NVS. For instance, the concepts of Neuroticism / Negative Emotionality in the Big Five model of personality [11, 12], Eysenck’s Neuroticism [13], Negative Affect [14, 15] and the Behavioral Inhibition System (BIS) in the Reinforcement Sensitivity Theory (RST; [16–19]) all encompass the processing of aversive emotional material. However, the degree to which these constructs are related to neurobiologically informed entities varies substantially, with Neuroticism being primarily defined based on self-report data, whereas the BIS and NVS conceptualizations are primarily defined based on neuroscientific evidence. Although the definitions of these different constructs may vary in detail, they all include the processing of aversive information and can therefore being considered components of the same underlying latent factor [20–22]. Accordingly, in the following these different conceptualizations are summed up with the term Neuroticism. Neuroticism has been conceptualized as a subordinate component of the general factor of psychopathology, which is evident by associations with various mental disorders, with particular relevance to internalizing disorders like depression and anxiety [23, 24].

Originally, reactivity of the amygdala in response to negative emotional stimuli has been proposed to underly Neuroticism [13, 25–29], but meta-analytic results did not corroborate these findings [30]. More recent evidence points to a negative association of Neuroticism with increased connectivity between the amygdala and the prefrontal cortex (PFC), reflecting the impaired ability of the PFC to regulate amygdala reactivity in individuals showing higher expressions of Neuroticism [31–36]. An integrative approach by Williams [37] has suggested dysfunctions in an affective network comprising the anterior cingulate cortex, medial PFC, insula, and amygdalae to be associated with negative affect. Also, the neurobiologically informed RST of personality [16–19] has stimulated considerable research regarding the involvement of the hippocampi in the processing of aversive states, particularly anxiety. Although promising biomarkers of Neuroticism have been identified, our understanding of the underlying neurobehavioral operating principles is still relatively scarce.

#### Rumination

Repetitive negative thinking is characterized by a diminished ability to disengage from spontaneous thoughts and poses a transdiagnostic risk factor with profound implications for mental health, mostly studied in the forms of Rumination and Worry [38–40]. Rumination has been assigned to the NVS in the RDoC framework [10] and is conceptualized as the repetitive and passive dwelling on causes and consequences of distressing events. It has been shown to be a risk factor for internalizing disorders in general and depression more specifically [41–46]. Rumination can be probed either as a trait, reflecting the propensity to use ruminative thinking in response to distressing experiences, or as a state, displaying the current proficiency of ruminative processes. Although often employed by individuals to cope with negative events, Rumination elicits opposing effects by mitigation of problem solving and instrumental behavior resulting in enhanced negative thinking and mood [42]. Worry, however, is characterized by a repetitive thinking about possible negative events in the future and has been primarily associated with anxiety [38]. Substantial correlations between self-reported Worry and Rumination [47, 48] as well as shared neural circuits [49, 50] have raised doubts whether they are to be considered independent processes with distinct psychopathological trajectories.

The default mode network (DMN; [51, 52]) is a large-scale neural network becoming active when no cognitively demanding tasks are carried out and is most commonly identified using resting state functional magnet resonance imaging (fMRI). Due to its implication in mind wandering and daydreaming, the DMN is proposed to resemble self-oriented internally focused spontaneous thought [40, 53, 54]. The DMN encompasses prefrontal regions, the anterior and posterior cingulate cortex, temporal regions, the hippocampi as well as parietal areas and angular gyri [37, 53–55]. Both, increased activity, and connectivity of the DMN have been reliably associated with Rumination and Worry during the resting-state as well as during Rumination induction tasks [49, 56–61]. Aberrant connectivities of the DMN, especially of the mPFC, have been consistently linked to depressive disorders [62, 63]. Moreover, the impaired ability to flexibly alternate between the DMN and task positive networks has been suggested to reflect the diminished ability to disengage from Rumination in depressed subjects [37, 60, 64–69].

#### Neuroticism-rumination-interaction

The interplay of Neuroticism and Rumination has primarily been investigated in the context of mediation models with Rumination mediating the relationship between Neuroticism and psychopathology [58, 70–76]. However, prior research has also pointed to Neuroticism and Rumination being related but distinct constructs, that exert effects on psychopathology independently from each other [63, 77]. Molecular approaches support this notion by showing that Neuroticism and Rumination are genetically related but separate over time in response to environmental influences [78, 79]. Although mediation models have been proven useful in elucidating the relationship of Neuroticism and Rumination, their distinctive and independent effects may be better represented in the context of moderation models. We therefore propose an amplification hypothesis: Neuroticism, here conceptualized as a distal higher-order vulnerability factor, exerts a direct effect on internalizing psychopathology. Rumination, here conceptualized as a dysfunctional coping mechanism and therefore more proximal risk factor, also exerts a direct effect on internalizing psychopathology independent from Neuroticism. If both coincide, their effects are hypothesized to potentiate and introduce additional risk for the respective person to develop internalizing symptoms, over and above Neuroticism and Rumination individually. Initial research suggesting direct as well as conditional effects of Neuroticism and Rumination fuels this hypothesis [80–83]. Additionally, a comparison of the moderation and mediation models to determine which model better explains the data could provide empirical evidence for the underlying nature of the association between Rumination and Neuroticism.

To our current knowledge, no neuroimaging studies have been conducted regarding the relationship of Neuroticism and Rumination. We therefore want to thoroughly elucidate their interrelatedness on the circuit as well as self-report level.

### The negativity bias in the context of cognitive emotion regulation and anhedonia

The Cognitive Systems [8, 9] domain in the RDoC matrix encompasses various cognitive processes, such as attention, perception, and working memory. Working memory, the executive ability to store and process information, and emotions have a reciprocal and modulatory relationship. Thus, the effects of emotions on working memory capacity as well as the influence of working memory on emotional experience have been highlighted [84]. Also, the effectiveness of cognitive emotion regulation and working memory performance are interconnected. On the one hand, this is attributed to the ability to update emotional information; on the other hand, working memory performance modulates the regulation of negative affect [85, 86]. Accordingly, reduced working memory capacity is associated with impaired cognitive emotion regulation [87]. In contrast, there is a positive relationship between working memory capacity and the use of adaptive emotion regulation strategies [88, 89].

Trait Negativity Bias is a transdiagnostic phenomenon that is relevant in affective, anxiety, schizophrenic, personality, and eating disorders [90–93]. This phenomenon is used to describe that negative emotional stimuli are more salient and, compared to positive or neutral stimuli, are processed preferentially [94]. In addition, faster reaction times are observed at the behavioral level, and increased reactivity of associated brain areas at the neural level [95, 96]. Effects of Negativity Bias are evident in attention, memory, and the interpretation of external information, among others [92, 97]. From an evolutionary perspective, the Negativity Bias underlies a formerly survival adaptive function [94], although it can affect mental health in maladaptive ways [98]. For example, in depressive spectrum disorders, Negativity Bias is associated with pathogenesis, maintenance, and recurrence [99, 100]. In numerous mental disorders, the Negativity Bias manifests itself with impairments in working memory performance in that negative stimuli are responded to more quickly and with a lower error rate than stimuli with positive or neutral valence [101]. This might be facilitated by reduced top-down cognitive regulation and increased activity of subcortical areas of emotion processing [99]. In contrast, an opposite effect of cognitive-affective processing has been reported in mentally healthy individuals, as stimuli with positive valence have been found to have improved accuracy and faster reaction times [102]. Independent of mental illness, associations of Negativity Bias with adverse childhood experiences and the personality trait neuroticism/negative emotionality have been highlighted [103–105]. For example, childhood neglect and maltreatment influence cognitive information processing in adulthood [106], and at higher levels of neuroticism, negative information is perceived more saliently than positive or neutral information [107].

At the neural level, during experimental working memory tasks, there is both increased functional connectivity within the frontoparietal or Central Executive Network (CEN) and increased activity in associated areas [108–110]. Primarily, this network is anchored in the lateral prefrontal cortex (lPFC). This again highlights the connection between working memory and cognitive emotion regulation, since, for example, functional connectivity between the lPFC and the amygdala is primarily relevant during positive reappraisal (Berboth & Morawetz, 2021), and the lPFC is thus involved in emotional self-regulatory processes. Based on meta-analytic evidence, Reid et al. [109] subdivide the CEN into an anterior and posterior part. Both are associated with components of cognitive control and working memory, respectively, with posterior areas more relevant to the maintenance of information and anterior areas more relevant to the further processing of that information [109]. Changes in functional connectivity within the CEN are related to the control of attention and emotion regulation. In this regard, hypo-connectivity within this network correlates with a deterioration of both [60]. In mental disorders, neuronal activity is reduced during maintenance, flexible updating, and interference control of information. This reduced activity is detected in areas directly associated with working memory, such as the lPFC and the anterior insula, as well as in the striatum [111]. Currently, there is no scientific evidence for the neural correlates of Negativity Bias in working memory, except that the lPFC is more activated when processing negative and positive information compared to neutral information [96]. However, a link between adverse childhood experiences and the Negativity Bias has already been highlighted, which is associated with increased activity in the pregenual anterior cingulate cortex [103].

The Positive Valence Systems [8, 9] are primarily responsible for responses to positive motivational situations or contexts, such as reward seeking, consummatory behavior, and reward/habit learning. Deviant reward processing is also a transdiagnostic phenomenon found in affective, schizophrenic, and post-traumatic stress disorders [112, 113]. Reward processes not only influence goal-directed attention [114] but also function as a possible top-down emotion regulation strategy [115]. Here, it is not only the initial hedonic response but more importantly the anticipation of reward [113]. For example, mental disorders are characterized by less salience of rewards and inadequate cognitive reactivation of rewarding past experiences [116]. At higher levels of Negativity Bias, rewarding stimuli are less salient and, moreover, are used in a reduced way for emotion regulation, which is associated with reduced reward anticipation [92, 117]. Hedonic capacity implies an individual's general response to appetitive stimuli and the motivation to achieve them. The negative manifestation of hedonic capacity is Anhedonia, i.e., the inability to feel pleasure or the diminished desire to engage in pleasurable activities [118]. Hedonic capacity can therefore always be evaluated in different contexts, such as the pleasure or lack of pleasure derived from sensory, social, and consumption-related events or the pursuit of hobbies. The transdiagnostic and typically treatment-resistant symptom of Anhedonia, has a central function in reward processing [119]. Anhedonia influences appraisal, anticipation, and motivation to reward, which is primarily associated with the ventral striatum at the neural level [120, 121]. The association between Anhedonia and poorer cognitive functions can be explained by reduced connectivity of reward-related neural areas [122].

Using a meta-analytic review of functional MRI studies, it was shown that significantly overlapping neural circuits are activated regardless of the type of rewarding stimulus, suggesting a general Reward Network (RN; [123]. Primarily, this network is anchored in the ventral tegmental area and ventral striatum. Reduced reactivity of the ventral striatum to rewarding stimuli is a transdiagnostic clinical biomarker [124–126]. Schwarz et al. [126] also highlighted that pathologically inhibited reward anticipation is associated with both reduced ventral striatum activity and reduced RN functional connectivity. This again clarifies the link between reward anticipation and Anhedonia, as ventral striatum activity acts as a neural biomarker in both [121, 123]. Moreover, increased ventral striatum reactivity during reward anticipation is associated with improved cognitive and social functioning, whereas decreased striatal activity is associated with increased affective lability [126]. Decreased reward reactivity is further related to stress-induced Anhedonia, adverse childhood experiences, and maladaptive coping strategies [127].

## Methods

### Hypotheses

#### Neuroticism

First, increased activation of the bilateral amygdalae and hippocampi as well as significant functional connectivity between the amygdalae and the PFC are hypothesized during negative emotional processing. These neural measures are hypothesized to be in turn associated with self-reported Neuroticism. Moreover, both self-reports and neural measures of Neuroticism are hypothesized to predict symptoms of internalizing psychopathology.

#### Rumination

Increased activation, and functional connectivity within nodes of the DMN are hypothesized to be observed during Rumination. Additionally, an association between these neural measures and self-reported Rumination is assumed as well as the prediction of symptoms of internalizing psychopathology by means of these measures.

#### Neuroticism-rumination-interaction

The interaction between Neuroticism and Rumination on the self-report and circuit level is hypothesized to predict symptoms of internalizing psychopathology. Moreover, the interaction terms of Neuroticism and Rumination on the self-report level are hypothesized to be correlated with the interaction terms of Neuroticism and Rumination on the circuit level.

#### Negativity bias

In working memory, negative and positive words are preferentially processed over neutral words. This is accompanied by an improvement in accuracy and reaction time and is also associated with increased activity and connectivity of the CEN and implicated areas, such as the lPFC.

#### Cognitive emotion regulation

The manifestation of Negativity Bias is associated with increased use of maladaptive cognitive emotion regulation strategies, such as Rumination, catastrophizing, and blaming oneself and others. Whereas positivity bias, i.e. preferential processing of positive stimuli, is associated with increased use of adaptive cognitive emotion regulation strategies, such as positive refocusing, reappraisal and refocusing on planning. Adaptive emotion regulation strategies are further related to increased activity and connectivity of the CEN and implicated areas, such as the lPFC.

#### Hedonic capacity

Negativity bias is positively related to Anhedonia, whereas positivity bias is associated with enhanced responsiveness to appetitive stimuli. Hedonic capacity is related to activity and connectivity of the RN and implicated areas, such as the ventral striatum, during reward anticipation. Anhedonia is associated with decreased activity and connectivity in these areas. Furthermore, a higher expression of the Negativity Bias inhibits the activation of the ventral striatum during reward anticipation. Finally, adaptive cognitive emotion regulation strategies are negatively related to Anhedonia.

### Participants

Based on a power analysis using G*Power [128], a sample size of *N* = 109 subjects is required to detect associations of moderate magnitude (*r* = 0.3), assuming an alpha threshold of 0.05 and a power of 0.90. Additionally, the sample size is increased by 10% to account for possible dropouts, resulting in a target sample of 120 complete data sets.

Only healthy young adults between 18 and 45 years will be enrolled in this study. Exclusion criteria include a diagnosed mental disorder, psychotherapy or psychopharmacological treatment in the past 12 months, pregnancy, or meeting any of the MRI exclusion criteria. Additionally, methylxanthines like caffeine have been shown to affect neuronal circuits involved in the processing of rewards [129], which is why participants who consume more than 8 standard caffeinated drinks per day are excluded from participation. Furthermore, subjects are instructed to refrain from alcohol and other psychoactive substances 48 h before the MRI assessment as well as methylxanthines at the day of the MRI assessments. Subjects are recruited using e-mail, personal approach, and advertising of the study in the online management system for psychological studies conducted at MSB Medical School Berlin and Free University Berlin. The study has been approved by the institutional review board of the MSB Medical School Berlin and all subjects provide written informed consent to participate. Psychology students at the MSB Medical School Berlin or Free University Berlin receive course credits for their participation in this study. Enrollment of participants started on 1^st^ of November 2022 and is currently ongoing. It is anticipated that the last subject will finish participation on 1^st^ of November 2023.

### Procedure

All subjects undergo two phases in this study. First, an online link is provided leading to an internet-based questionnaire (www.soscisurvey.de) to determine the eligibility of potential participants. In that vein, written informed consent to participate in this study is provided by the subjects, followed by the Mini DIPS [130] to ensure subjects do not currently suffer from mental disorders or have been in treatment for a mental disorder in the past 12 months. Moreover, employing a check list provided by the MRI site, subjects fulfilling any of the MRI exclusion criteria are denied further participation in the study for safety reasons.

After successfully determining the eligibility of the subjects, a second online link is provided to complete questionnaire set 1 encompassing a variety of constructs related to psychopathology, transdiagnostic phenomena and other variables of interest. The respective section below gives a full description of the questionnaires in use. Questionnaire set 1 takes about 60 min to complete.

Third, subjects receive an appointment for the MRI measurements taking place at the Center for Cognitive Neuroscience Berlin. After arrival, participants first complete additional self-reports for about 60 min, followed by the MRI assessment.

**Fig. 1:**
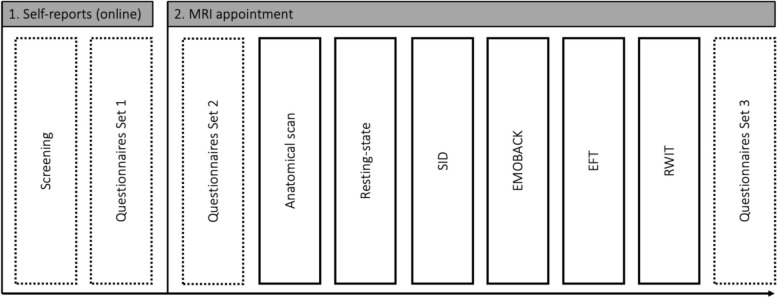
Procedure SID, Social Incentive Delay Task; EMOBACK, Emotional N-back Task; EFT, Emotional Faces Task; RWIT, Rumination and Worry Induction Task

During the MRI assessment, first, an anatomical scan is conducted followed by a resting-state sequence. The resting-state measurement is implemented as the first functional sequence to rule out carry-over effects elicited by emotionally valenced material from the tasks. Then, the SID task [131] and the EMOBACK task [96, 132] are used to investigate neural responses to social reward anticipation and cognition-emotion interactions, respectively. Subsequently, the Emotional Faces Task employing human faces expressing negative emotions, which has been shown to robustly activate the bilateral amygdala [96], is used to probe the processing of negative emotional stimuli in the brain. Lastly, the Rumination and Worry Induction Task (based on [58, 59, 133–135]) is employed to investigate brain activation and connectivity elicited by Rumination and Worry, respectively. Total duration of the MRI examination is 1.75 h and after completion, participants fill out questionnaire set 3 (duration 2 min). A visual depiction of the procedure can be found in Fig. 1.

### Questionnaires

Various questionnaires are employed to assess personality traits, coping styles, hedonic capacity, constructs related to psychopathology and covariates like socioeconomic status and need for cognition. An overview of the questionnaires is given in Table 1.

**Table 1:** Questionnaires

Questionnaire	Description	Set
*Personality*		
**Adult Temperament Questionnaire** (ATQ; [14, 136])	Constructs: negative affect, effortful control, extraversion, orienting sensitivity 77 items	Set 1
**Action Regulating Emotion Systems** (ARES [137];)	Constructs: behavioral inhibition system sensitivity, behavioral activation system sensitivity 20 items	Set 1
**Big Five Inventory 2** (BFI-2 [12, 138–140];)	Constructs: negative emotionality, extraversion, agreeableness, conscientiousness, openness to experiences Only the negative emotionality domain from the long version of the BFI is used: 12 items; as well as the full short version: 21 items	Set 1
**Behavioral Inhibition System / Behavioral Activation System Scales** (BIS/BAS; [141, 142])	Constructs: behavioral inhibition system sensitivity and behavioral activation system sensitivity 24 items	Set 1
**Sensitivity for Punishment and Sensitivity for Reward Questionnaire (SPSRQ** [143, 144]**;**)	Constructs: sensitivity for punishment, sensitivity for reward 48 items	Set 2
**Positive and Negative Affect Schedule** (PANAS; [15, 145])	Constructs (trait, state): positive affect, negative affect 20 items	Set 1, Set 2, Set 3
**Reinforcement Sensitivity Theory of Personality Questionnaire** (RST-PQ; [146, 147]	Constructs: behavioral inhibition system sensitivity, behavioral activation system sensitivity, fight-flight-freeze system sensitivity 65 items	Set 1
*Emotion regulation*		
**Perseverative Thinking Questionnaire** (PTQ; [148]	Construct: repetitive negative thinking 15 items	Set 1
**Response Styles Questionnaire** (RSQ; [149–151])	Constructs: rumination (brooding, reflection), distraction 37 items	Set 1
**Brief State Rumination Inventory** (BSRI; [152])	Construct: rumination 8 items	Set 2, Set 3
**Penn State Worry Questionnaire** (PSWQ; [153, 154])	Construct: worry 16 items	Set 1
**Cognitive Emotion Regulation Questionnaire** (CERQ; [155, 156])	Constructs: self-blame, blaming others, acceptance, refocus on planning, positive refocusing, rumination, positive reappraisal, putting into perspective, catastrophizing 36 items	Set 2
**Five Facets of Mindfulness Questionnaire** (FFMQ-D; [157, 158])	Constructs: mindfulness, describe, act with awareness, non-judging, non-reactivity, observe 39 items	Set 1
*Hedonic capacity*		
**Snaith Hamilton Pleasure Scale** (SHAPS; [159, 160])	Constructs: Anhedonia in social interaction, eating and drinking, sensory experiences, interests/leisure activities 14 items	Set 2
**Leuven Affect and Pleasure Scale** (LAPS; [161])	Constructs: negative affect, positive affect, hedonic tone, cognitive functioning, overall functioning, meaningful life, and happiness 16 items	Set 1
**Dimensional Anhedonia Rating Scale** (DARS; [162, 163])	Constructs: desire, motivation, effort and consummatory pleasure across hedonic domains (hobbies, food/drink, social activities and sensory experiences) 17 items	Set 2
*Psychopathology and stress*		
**Mini Diagnostic Interview for Psychiatric Disorders** (Mini DIPS; [130, 164])	Constructs: Screening for mental disorders by means of the ICD-10, past diagnosis or treatment of mental disorders	Screening
**Depression, Anxiety and Stress Scale** (DASS; [165–167])	Constructs: symptoms of depression, anxiety, stress. The depression and anxiety dimension are summed up to form a scale comprising internalizing psychopathology 42 items	Set 2
**State Trait Anxiety Inventory** (STAI; [168, 169])	Constructs: state anxiety, train anxiety 40 items	Set 2
**Perceived Stress Scale** (PSS10; [170, 171])	Constructs: chronic stress: helplessness, self-efficacy 10 items	Set 2
**Trier Inventory of Chronic Stress** (TICS; [172])	Constructs: chronic stress: work overload, job dissatisfaction, social stress, lack of social recognition, worry/concern, stressful memories 57 items	Set 1
**Childhood Trauma Questionnaire** (CTQ; [173, 174])	Constructs: childhood maltreatment, emotional neglect, emotional abuse, physical neglect, physical abuse, sexual abuse 28 items	Set 1
*Other*		
**Need For Cognition** (NFC; [175])	Construct: pleasure in thinking tasks 33 items	Set 1
**Subjective Social Status** (SSS; [176, 177])	Constructs: local and national related subjective social status 2 items	Set 2

### FMRI paradigms

#### Resting-state

During the 10 min resting-state, subjects are instructed to relax, to not think about anything in particular and let the mind wander, while looking at a fixation cross.

#### Social Incentive Delay Task (SID)

The SID task [131] investigates reward anticipation and consists of 88 blocks, that last approximately 10 min. Within each block, a possible reward is dependent on the subject's reaction time. This is evaluated in advance to allow for individual variability. At the beginning of a block, a cue stimulus is used to announce the possible reward, thus eliciting reward anticipation. This is done by presenting a circle with 1, 2, or 3 horizontal lines for 240 ms. The more lines the circle contains, the higher the potential reward. As a control condition, the presentation of a triangle serves as a cue that no gain can be obtained in this block, regardless of the reaction time. The cue stimulus is followed by a pause of 2250–2750 ms, in which a crosshair is presented. Subsequently, a filled square is presented as the target stimulus for 160–260 ms (adapted to the individual reaction time) and the participants are asked to press a key as quickly as possible. Depending on the reaction time (with a cut-off at 66%, i.e., the key press must occur within 111–172 ms), the rewarding stimulus (1650 ms) follows after an ISI of 300 ms. This represents, depending on the cue stimulus and reaction time, a face with an increasingly happy expression (3 intensity levels). Faces are randomly sampled from a pool of 66 photos (22 different subjects (11 female, 11 male) with happy facial expressions of three differing intensities). Photos from the NimStim set of Facial Expressions database are used for this purpose (available at http://www.macbrain.org; [178]). If the reaction time was below the individual cut-off value, or the cue stimulus of the block was a triangle and thus a "no win" stimulus, only an unrecognizable face is presented using morphing software (see [131]).

#### Emotional n-back Task (EMOBACK)

This is an approximately twelve-minute n-back task using emotional words from the Berlin Affective Word List (BAWL; [179]). Words are classified as positive, negative, or neutral according to BAWL norms, with 35 different words assigned to each valence. All words are nouns and comparable in terms of length (5–8 letters), figurativeness, and frequency (total frequency of occurrence per million words). During the task, stimuli are presented block by block via a screen. Participants are asked to respond by pressing a key when the currently presented word matches the three-to-one (3-back). The blocks each consist of 15 words of one of the three valences and are presented for 500 ms with a 1500 ms inter-stimulus interval (ISI). Between blocks, there is a pause of 10–14 s during which a crosshair is seen on the screen. A total of 75 words are presented per condition. The order of the blocks is randomized.

#### Emotional Faces Task (EFT)

To investigate functional brain changes during emotional challenge, subjects are presented faces expressing the negative emotions fear, sadness, and disgust from the Warsaw Set of Emotional Facial Expression Pictures [180]. First, a brief training of the task is completed inside the scanner in advance of the data acquisition. During the task, a total of 72 emotional faces (counterbalanced for sex) are shown for 3 s each and scrambled pictures showing random color patterns with either a blue or a green frame are employed as the control condition. Subjects are instructed to report the sex of the depicted person or the color of the frame using button presses, to ensure sufficient task engagement. A total of 12 blocks with faces and scrambled pictures are presented in randomized order, with each block lasting for 18 s. During the inter-trial interval, a white fixation cross on a dark background is shown. Total task duration is 13 min.

#### Rumination and Worry Induction Task (RWIT)

Rumination and Worry are induced experimentally using the RWIT. In contrast to most rumination induction tasks [58, 59, 134], the RWIT aims to not only elicit Rumination, but also Worry. Since recent studies have proposed a relationship between Rumination and negative mood in HV [58, 181]. Hence, participants complete a dysphoric mood induction procedure prior to the start of the RWIT, by recalling a situation, which elicited negative emotions like guilt, shame, sadness, or fear, and vividly remembering this situation for 2 min. Next, participants are instructed to think about the negative event by means of self-oriented questions proposed to elicit Rumination and Worry, respectively. For instance, an item proposed to elicit Rumination is “Why can't I handle events like this better?” and for Worry “How can the event affect my future negatively?”. As contrast condition, subjects are shown 4 brief sentences describing a situation and instructed to vividly imagine it, for example “A typical classroom from school”. Prior to every condition, a slide is shown on the screen for 30 s to explain the goal of the following condition. Each condition includes 4 questions and sentences, which are shown for 90 s. The Rumination and Worry condition are presented in a randomized order, whereas the control condition is always presented last to ensure that the experimental conditions are undergone continuously. In total, the RWIT takes 20 min.

### MRI acquisition and analysis

#### MRI acquisition

Brain images are acquired using a 3 Tesla MRI scanner (PRISMA fit, Siemens Medical Systems, Erlangen, Germany) and a 32-channel coil. The anatomical image is acquired by means of a 3D T1 weighted sequence (Magnetization Prepared Rapid Acquisition Gradient Echo sequence, TR = 2.3 s, TE = 3.03 ms, slices = 192, voxel size = 1 × 1x1 mm, flip angle = 9°, FOV = 256 × 256x192 mm). Functional brain images are acquired using a T2* weighted gradient echo-planar imaging sequence sensitive to the BOLD effect (Blood Oxygen Level Dependent), accelerated by a factor of 3 (TR = 1 s, TE = 0.3 ms, slices = 39, voxel size = 3 × 3x3 mm, flip angle = 65°, matrix = 64 × 64, FOV = 192 × 192x140 mm, MB factor = 3). Moreover, to improve registration a fieldmap is obtained employing a double-echo gradient echo field map sequence (TR = 468 ms, TE = 4.92 / 7.38 ms, slices = 39, voxel size = 3 × 3x3 mm, flip angle = 60°, matrix = 64 × 64, FOV = 192 × 192x140).

#### MRI analysis

Brain image preprocessing and analysis are carried out using FEAT (FMRI Expert Analysis Tool; [182–184]) version 6, as part of FSL (FMRIB’s Software Library; [185–187]).

##### Regions of Interest (ROIs)

The bilateral amygdalae, hippocampi as well as the PFC are defined as regions of interest for the main analyses of the EFT. During the RWIT, seeds within the DMN are defined as ROIs, specifically the posterior cingulate cortex as seed for the core-system and the hippocampi as seed for medial-temporal subsystem. ROIs related to the SID task for anticipation are the ventral striatum, dorsal anterior cingulate cortex, supplementary motor area, anterior insula, and ventral tegmental area. During the initial hedonic response to social reward, the dorsal caudate, supplementary motor areal, anterior insula, putamen, thalamus, and midbrain are relevant. For the EMOBACK task, the focus is on the lateral prefrontal cortex (up to BA45 & BA44), posterior medial prefrontal cortex, intraparietal cortex, anterior insula, and inferior frontal gyrus. For the connectivity analyses, both SID and EMOBACK ROIs are extracted from the ANIMA database [109]. For SID, the Reward Network [123], and for EMOBACK, the Central Executive Network [188].

##### Preprocessing

T1 anatomical data are biasfield corrected and aligned to the MNI152 standard space using linear alignment via FSL FLIRT with 12 degrees of freedom and subsequently refined non-linearly as implemented in FSL FNIRT. The processing of the functional brain images includes correction for participant head motion, correction for EPI distortions using fieldmap data, and a 5 mm FWHM spatial smoothing. To identify and correct for more subtle effects of head motion and noise induced by the multiband acceleration or physiological processes, ICA-based methods are used. Finally, a temporal 0.01 Hz high-pass filter is applied to remove scanner drifts. Furthermore, a transformation from the functional space to the T1 anatomical space using FSL Boundary Based Registration is obtained. Eventually, the transformation is combined with the T1 to MNI152 registration to transfer the functional data from the individual’s native space to the MNI152 standard space.

##### First-level

A General Linear Model implemented in FEAT is used on the preprocessed participant-level data in combination with Python [189] to obtain the contrasts of interest. First, the onsets of the respective task conditions are calculated from the log files and subsequently used by FEAT to model the task-specific BOLD signal changes. Thereafter, an estimate of the fit between the task regressor and the BOLD time series is obtained for each voxel, ultimately resulting in a map of statistically significant activations. Multiple comparisons correction is performed at the cluster level using Gaussian Random Field Theory (voxel: z > 3.1 uncorrected; cluster: *p* < 0.05, corrected). The resulting activation maps are used in subsequent group-level analyses. Moreover, to investigate functional connectivity between regions of interest and the rest of the brain during the tasks, a psychophysiological interaction analysis is conducted. The respective regressor is acquired by forming an interaction term of the time series of the voxel with the best fit to the task regressor in a given ROI as the seed and the task regressor of interest. Applying this new interaction regressor using the General Linear Model described above results in a connectivity map of regions significantly correlated with the seed region.

##### Group-level

A General Linear Model using FSL will be implemented to tests for statistically significant activations and connectivities. Multiple comparisons correction is performed at the cluster level (voxel: z > 3.1 uncorrected; cluster: *p* < 0.05, corrected). Moreover, ROI analyses are conducted using FSL featquery to extract BOLD percent signal changes and contrast of parameter estimates, to be used in later stages of the analysis pipeline.

##### Resting-state

For the analysis of resting-state functional connectivity the CONN toolbox [190] will be used to obtain seed-based correlation maps on the whole-brain level as well as connectivity measures between regions of interest to be used in later stages of the group-level analyses.

### Statistical analysis

All statistical analyses going beyond the image processing will be conducted in R [191, 192] or Python libraries such as Pandas [193], Matplotlib [194], and SciPy [195]. Multiple comparisons correction is performed using the FDR method assuming α = 0.05.

#### Neuroticism-rumination-interaction

First, to represent the higher order nature of Neuroticism, structural equation modeling is used to generate a latent variable using the respective scales of the ATQ, BFI, PANAS trait as well as the ARES, SPSRQ, BIS/BAS, and RST-PQ. Subsequently, the results of the SEM are used on the individual level to remove variance from the data not specific to the latent variable, resulting in a denoised measure of Neuroticism.

Next, hypotheses are tested statistically using regression models adjusting for age and sex. The association of self-reported Neuroticism with amygdala and hippocampus reactivity as well as the functional connectivity of the amygdalae to the PFC during the EFT are tested using multiple regression. In that vein, the effect of Neuroticism as a generic risk factor for internalizing disorders is tested using linear models, including self-reported and neural measures of Neuroticism as predictors of internalizing symptomology as the dependent variables. A measure of internalizing symptomology will be obtained by calculating a standardized composite of the depression and anxiety scales of the DASS.

Similarly, the association between self-reported Rumination and DMN connectivity during the RWIT is tested by employing linear models. Additionally, the effect of Rumination as a transdiagnostic risk factor is evaluated using multiple regression, including self-report and neural measures of Rumination as predictors and internalizing psychopathology as the dependent variable.

Ultimately, a moderator analysis is used in the context of a multiple regression to investigate the interaction effect of Neuroticism and Rumination on internalizing psychopathology. Herein, the interaction term is formulated for the self-report and neural measures, respectively. Furthermore, an association between the interaction terms of the self-report and neural level is analyzed.

Exploratorily, the effect of Worry as a transdiagnostic risk factor is investigated. As already described in the introduction section, Worry is a cognitive process closely related to Rumination. Therefore, the analyses described above are repeated using Worry instead of Rumination to elucidate the interaction of Neuroticism and Worry on internalizing psychopathology. Additionally, DMN connectivity estimates derived from the resting-state instead of the RWIT are employed as neural measures of Rumination and used in the respective subsequent analyses. Finally, a model comparison approach is planned to determine whether the data is explained better by a moderation or a mediation model regarding the relationship between Neuroticism, Rumination, and psychopathology.

#### Negativity bias

To calculate a value for the negativity or positivity bias, the behavioral data of the EMOBACK task are utilized. The average accuracy and reaction time is used by calculating the difference between the negative and positive condition. These values will be z-standardized. A positive value implies the presence of a Negativity Bias, and a negative value implies a positivity bias. In the next step, the standardized values for accuracy and reaction time are examined for correlation with the functional neural data of the CEN during EMOBACK.

#### Cognitive emotion regulation

Neural data from the activity and connectivity analyses of the CEN especially the lPFC during the working memory task will be correlated with the two main scales of the CERQ (adaptive & maladaptive cognitive emotion regulation strategies). In the following step, multiple regressions are performed, with the subscales of the adaptive and maladaptive strategies as predictors. Similarly, the association between Negativity Bias and cognitive emotion regulation strategies will be tested.

#### Hedonic capacity

Neural data from the activity and connectivity analyses of the RN especially the ventral striatum during the social reward anticipation task will be first correlated with hedonic capacity/tone main scales from LAPS and DARS questionnaires. In addition, linear regressions will be used to examine the relationship of activity/connectivity with the subscales of the LAPS (cognitive functioning, overall functioning, meaningful life, and happiness) and those of the DARS (social activities, sensory experiences, hobbies, and food/drink). Similarly, the association between Negativity Bias and hedonic capacity variables will be tested.

## Discussion

Internalizing disorders are the most common type of mental disorders with substantial impact on public health [1, 5]. Many of these disorders are characterized by alterations in both cognitive and emotional processes [196]. In the past, cognitive and emotional processes associated with psychopathologies were investigated within the boundaries of phenomenologically based diagnostic criteria. However, recent developments highlight the need for a dimensional approach, rooted in multimodal assessment of neurobiologically informed entities of neurobehavioral functioning [8, 9]. Transdiagnostic phenomena such as Neuroticism, Rumination, Anhedonia, the Negativity Bias and maladaptive emotion regulation strategies are known to interact with each other, however current findings are contradictory and the exact nature of their relationships remain to be unknown [197]. This study aims to collect data on the circuit, behavioral and self-report level to investigate the operating principles of the neurobehavioral processes associated with psychopathology mentioned above.

A better understanding of the mechanisms underlying the relationship between Neuroticism and Rumination as well as hedonic capacity, Negativity Bias and emotion regulation strategies may guide future development of diagnostic labels and treatments.

Limitations of the present study include the cross-sectional design, which renders inferences to be correlational, so no causal conclusions regarding the relationship between the respective constructs of interest can be drawn. Since the sample is restricted to currently healthy subjects, no conclusions can be drawn regarding diagnosable mental disorders and limits the interindividual variability of the psychopathological phenomena in question. However, the requirements regarding the mental health of the sample are considerably low. Subjects are only required to not fulfill the criteria for diagnosis of a mental disorder or receive professional mental health interventions in the past 12 months to take part in this study. Since, diagnosis and treatment for mental disorders that lie further in the past are not taken into account and subsyndromal symptoms of mental distress are not sufficient to exclude subjects from participation. Therefore, measures of psychopathology are expected to show variability, at least to some extent.

## Data Availability

Not applicable.
